# Antiseptic Surgery

**Published:** 1890-05

**Authors:** E. T. Lawley-York

**Affiliations:** Ontario Veterinary College, Toronto


### ANTISEPTIC SURGERY.[1]

BY E. T. LAWLEY-YORK,
*Ontario Veterinary College, Toronto.*

A GREAT change has taken place in the practice of surgery since the year 1860. This change is due to the fact that the germ theory of disease has been accepted by the great majority of surgical teachers and practitioners. Scientific men have demonstrated that the causation of many diseased conditions is closely connected with the presence in the diseased organ, tissue, or individual, of living organisms, which have to a certain extent been classified, and are supposed to be forms of plant life.

It has long been known that subcutaneous injuries follow, as a rule, a very different course from open wounds, and the past history of surgery gives evidence that surgeons not only were aware of this great difference, but endeavored by the use of various dressings empirically to prevent the evils which were matters of common observation during the healing of open wounds.

Various means were adopted to prevent the entrance of air, *e. g.*, in the opening of abscesses by the valvular method of Abernethy ; and by the subcutaneous division of tendons in the common disease termed "club foot." Balsams, turpentines and various forms of spirit were the basis of many varieties of these dressings.

In the beginning of the Nineteenth Century those complicated dressings began to lose favor, and practical surgeons went to the opposite extreme, using only the most simple dressings, the main object of which was to allow a free escape of discharge. Others applied no dressings at all, simply laying the stump of a limb after an operation on a piece of dry lint ; others again left the wound open for some hours after an operation, preventing in this way any accumulation, and brought its surfaces and edges together after all oozing of blood had ceased.

As a result of these various improvements many wounds healed in a thoroughly satisfactory manner. But in other cases

[1] Read before the Veterinary Medical Association of the Ontario Veterinary College, March 11th, 1890.

inflammation often occurred, accompanied by pains and suppuration or the formation of pus, and various feverish conditions due to, and in some way connected with, the unhealthy state of the wound were observed.

These constitutional sequelæ frequently proved fatal, and the general impression of surgeons was either that the constitution of the patient rendered him liable to these conditions, or that some poison had entered into the wound, and passing from it into the veins or lymphatic vessels that had been cut across reached the general circulation, contaminating the blood and poisoning the patient. The close clinical association between suppuration (or the formation of pus) in wounds and many of those fatal cases encouraged the belief that the pus cells from the wound entered the circulation (whence the word "pyæmia"). It was also frequently observed that a septic condition of the wound was associated with the constitutional and it was supposed that the septic matter passed into the blood (whence the word "septicæmia"). It was further observed that the crowding together of patients with open wounds increased the liability to those constitutional disorders. It is generally believed that before the advent of anæsthetics and antiseptics death was invariably due after an operation either to shock or blood poisoning.

This was very much the state of affairs when Lister, in 1860, from a study of the researches of Pasteur into the cause of putrefaction, stated that the evils observed in open wounds were due to the admission into them of organisms which exist in the air, in water, sponges, instruments and on the hands of the surgeons. These organisms, finding a suitable nidus for their growth and development in the discharges and surrounding tissues, germinate in them and alter their chemical constitution, forming various poisonous compounds which if absorbed into the blood give rise to pyæmia and septicæmia. He had to deal with a germ, and he desired to interfere with its growth. This was possible in one of two ways: Directly destroying or paralysing the germ itself before it had entered the wound, or after it had, or by an interference with the soil in which it grew, for example, by facilitating the removal of the discharges and preventing their accumulation in the wound cavity; and by doing everything to prevent depression of the wounded tissues, because healthy tissues are the best of all germicides.

Several substances were then known as possessing properties antagonistic to sepsis or putrefaction, and hence called antiseptic. Lister chose for his experiments carbolic acid, which he used at first in a crude and impure form. He had many practical difficulties to contend with—the impurity of the substance, its irritating properties, the difficulty of finding the exact strength in which to use it, fearing lest it should be too strong it might irritate the tissues to which it was applied; on the other hand he feared to use it too weak, lest its antiseptic properties should be insufficient for the main object in view. After many disappointments he gradually perfected his method of performing operations and dressing wounds.

*The Germ Theory of Disease.*—That putrefactive suppuration is a species of fermentation produced by microscopic-organisms, is a well-established fact. Time will not allow of my going minutely into their classification, but those wishing to do so will find "Crookshank's Introduction to Practical Bacteriology" a most useful aid to its study.

Prof. Williams, in his "Veterinary Medicines," page 811, 3d edition, gives a short and good description of them. During the past few years there has been a great controversy among surgeons in reference to the cause of septic infection in surgical wounds. Some claim that the micro-organisms are present in the air, and that if these can be prevented from coming in contact with the wound no decomposition or infection can occur. Others again claim that sepsis is not caused by micro-organisms in the air, and that air itself in contact with a wound will not cause decomposition. For the sake of convenience the following divisions may be made:

I. The presence of micro-organisms.

II. Their action upon living tissue.

Micro-organisms may be present in the body independently of any disease. The mucous membrane, the respiratory organs, are covered with millions of micro-organisms, although the deeper tissues escape; but should the deeper tissues become impaired or a secretion altered, then those dreaded agents appear.

From experiments made on shipboard, micro-organisms are entirely absent at from 70 to 120 miles from land. If we consider for a moment that the air contains millions of those micro-organisms in which daily operations are performed, and the fact is demonstrated that these germs cause putrefaction, in fact, are the unseen cause, the thing is how best to protect our patients against the forces that can turn the battle of life or death in an operation.

Micro-organisms consist of cellulose membrane, protoplasm, cilia and flagella.

Cellulose membrane is impervious to the action of acids or alkalies, although very thin.

Protoplasm is generally colorless and always nitrogenous.

Cilia or flagella are found in certain forms of bacteria and bacilli.

Micro-organisms require warmth, nutrition, moisture ; 98° F., or blood heat, is found to be most favorable ; great extremes are fatal, viz., freezing or boiling, which of course cannot be applied to surgery.

Nitrogen is essential. It may come from the albuminous compounds through the process of putrefaction, or from nitric acid in the ammonia. Carbon is also necessary ; oxygen is necessary to some and fatal to others.

Water is absolutely necessary ; without it the movements of bacteria and bacilli are arrested.

As regards their action upon living tissues. Should any of these germs fall upon an unprotected wound, so sure will decomposition take place. Thus it is of the greatest importance that the surgeon should take care in the selection of his antiseptic dressings, to also well study the proper time to renew them, as meddlesome interference is also productive of great mischief.

*Theory of the Formation of Pus.*—It is a matter of no difficulty to detect by appropriate methods the presence of micrococci in all collections of pus, large or small, in the animal body. It is a matter of more difficulty to discriminate the various forms met with. The following species have been discriminated :

Streptococcus pyogenes.
Staphylococcus pyogenes aureus.
Staphylococcus pyogenes albus.
Staphylococcus pyogenes citreus.
Staphylococcus cereus albus.
Staphylococcus cereus flavus.

The staphylococcus pyogenes aureus is perhaps the most generally distributed of all pyogenic or suppurative bacteria. It is called aureus on account of the golden yellow color of the cultivations. It grows in chains, but generally forms masses or colonies. It grows rapidly and has great power of resistance, and may retain its vitality for some months ; the best temperature for it is from 86° to 100° F. It can grow with little or no air, but the presence of oxygen is necessary. This organism is found in nearly all collections of pus, in boils, carbuncles, pustules of skin (impetigo) and abscesses of the subcutaneous tissues. It is important to notice that the same organisms may be obtained from cultivation from the surface of the body, in moist parts of the skin, especially if dirty, and in matter from under the nails, and has been found in healthy mucus from the pharynx and normal saliva. Further, it has been found apart from the human body in "slop" water of kitchens, in the earth, and even lately, it is said, in the air.

There is, therefore, great probability that it is widely scattered about in human dwellings, and must be regarded as having an ectogenic as well as a parasitic life.

There can be no doubt that this organism is a direct producer of suppuration, and is able alone to set up this process. For proof it is hardly necessary to go beyond the experiments which several pathologists have made upon themselves.

It may be asked, if the organism is so frequently present on the surface of the body, why does it not oftener produce suppuration ?

The reason seems to be : First, that some kind of wound or considerable pressure is necessary to enable the cocci to penetrate the skin ; secondly, that some skins, for instance those of children, are more easily penetrated, and thus children are particularly liable to impetigo and to "festering" wounds ; and, thirdly, there must be conditions, not clearly definable, which makes the tissues of some persons, and of the same person at different times, especially liable to the attacks of bacteria or deficient in power of resistance.

The actual process by which staphylococcus sets up suppuration seems to be chiefly by its solvent and necrotic action on the tissue elements ; and by the injury thus caused to the walls of the bloodvessels, which then permit increased transudation and emigration of leucocytes.

It may be plausibly supposed that some enzyme or ferment generated by the bacteria is the actual solvent; but no such substance has yet been isolated.

That the various pyogenic organisms already spoken of are actually the cause of suppuration cannot be doubted; but it is certain that this process can be set up by other agents.

For instance, powerful chemical irritants, such as turpentine, croton oil, carbolic acid, ammonia, when injected into subcutaneous tissue of animals, with strict precautions to prevent the entrance of bacteria, have produced abscesses; and it is to be observed that some of the aforementioned substances are powerful bactericides, and would prevent the growth of an organism.

*What It Has Done for Operative Surgery.*—Within recent years the main advance in surgery has been from the scientific side, due to the increased precision in physiological knowledge and a careful study of the relations of organisms to diseased conditions. And with this progress operative skill, in many directions previously unthought of, has kept pace.

The abdomen, long the exclusive province of the physician, has now become one of the favorite domains of the surgeon, where he has won some of his most amazing triumphs.

The growing relative popularity and success of surgery are, no doubt, due to the fact that its methods admit of greater precision than those of medicine. There is no more interesting study in surgical history than the development of our present practice as regards wounds, from the blind gropings of the surgeons of last century to discover the secret enemies that baffled their best efforts to the brilliant dawn we have been permitted to see.

The proper use and best form of deep and superficial stitches and dressings are now well established, and the reign of dirty sponges, foul instruments and hands has passed away forever.

We now come to what will ever be looked upon in future time as the commencement of a new era in surgery.

In 1847, at Derby, Mr. Walshe, of Worcester, introduced, for the first time, the subject of "Anæsthesia!" I would merely allude to this casually, being, as it is, so intimately connected with modern operative surgery.

At a meeting of the British Medical Association, in 1862, Paget spoke most eloquently of the management of patients after surgical operations, and urged upon all the study of the large group of diseases classed under the name of *pyæmia*, their origin, multiform nature and mode of prevention. It was the despairing cry of a great surgeon, who felt the deep regrets and bitter disappointments from which we might be saved, were there less risks, after the many operations to save life. The object Lister had in view, from the beginning of his experiments, was to place the open wound in a condition, as regards the entrance of organisms, as closely analogous as possible to subcutaneous wounds, such as a contusion or simple fracture, in which the unbroken skin acts as a protection to the wounded tissues beneath.

The introduction of the practice by Lister effected a complete change in operative surgery. Surgeons now understood the dangers which lay on every side, and this knowledge causes them to take greater care in the putrefaction and in securing greater cleanliness of wounds.

Antiseptics were originally substances which interfered with sepsis; the term has now a wider meaning, and includes any substance opposed to fermentation. "Antifermentative," or antitheric, would be a better term. An antitheric substance is one which interferes with fermentation by destroying or paralyzing the organisms which are the primary cause of the condition. On the other hand, the word antiseptic would be reserved to denote any substance which is opposed to putrefaction or sepsis—one form of fermentation.

Many of the most dangerous fermentations have nothing in common with putrefaction. The products which result are odorless; the appearances which arise bear no similarity to the changes which occur when putrefactive fermentation is present. The fermentations of smallpox, syphilis, scarlet fever, typhoid, relapsing fever, typhus, erysipelas and cholera may be taken as fermentations of the non-putrefactive class. Apparently in them the organisms enter the blood stream, there develop, and form their products, which, acting directly or indirectly on the heat centre, give rise to a specific fever. This fever continues until the soil is worn out, and the organism, finding no longer a nidus for its development, dies out, and recovery takes place.

Death, of course, results if the animal has not sufficient strength to withstand the attack. An attempt has been made to divide into two great divisions the effective and the non-effective. The first class can grow upon living tissue, the second class cannot; the first form their products upon living matter, the second can only grow in dead or lowly-vitalized matter.

The infective organisms can migrate from their original point of entrance, by the vascular and lymphatic streams, to distant parts of the body, and may there form secondary foci of infection. As regards the non-infective, the manufactory of the poison is principally restricted to the near neighborhood of the original point of entrance, generally a wound; it cannot migrate into the living tissues around if they remain healthy.

*Operations in which It Can Be Applied.*—How can we profitably apply antiseptics to every-day operations? This is a very difficult question to answer. Of course, we cannot possibly observe the strict cleanliness that is observed in a hospital, but much can be done by the judicious use of antiseptics in a great many operations, viz., poll evil, fistulous withers, removing shoe boils, spaying, quittor, punctures and pricks, castrating, etc.; also by endeavoring to observe a few of the following rules:

The hands and arms, especially the finger nails of the surgeon and his assistants, should be well scrubbed; rings, especially those having stone settings, should never be worn during operations.

Instruments should be subjected to a careful and minute cleansing with soap and brush, care being taken to remove all dry particles of pus, blood, etc., from the grooves; they should be immersed in a 3 per cent. solution of carbolic acid before use.

Wound Irrigation.—During the operation the wound should be frequently irrigated with a proper kind of disinfecting fluid, also the hands of the surgeon should be washed with it at not too long intervals (corrosive sublimate, 1 part to 1000). The instruments should also be kept in a 3 per cent. solution of carbolic acid, which is least injurious to them.

Sponges should be beaten free from all calcareous substances and immersed for fifteen minutes in dilute muriatic acid, to dissolve any remaining lime; then immersed in a 5 per cent. solution of carbolic acid, in which they remain until required for use.

Materials for Ligatures and Sutures.—The simplest way of preparing catgut is Kocher's. Immerse it for twenty-four hours in good oil of juniper—oil of the berry, not wood—transfer and preserve in alcohol until wanted; alcohol keeps it hard and firm, yet flexible; carbolic acid weak and brittle. Silk can also be rendered unirritant by boiling it an hour in a 5 per cent. solution of carbolic acid, then preserving in alcohol. Drainage tubes and elastic ligatures are kept in a 5 per cent. solution in large, wide-mouthed, stoppered bottles.

Types of Dressing.—Certain finely powdered substances, as iodoform, boracic acid, have the quality of rapidly inspissating blood and serum to a dry crust. Accordingly, after the hemorrhage has been controlled and the wound closed by a suture, a quantity of the substance chosen is dusted over. No further dressings are applied ; the escaping bloody serum forms a paste with the powder which, by its sterilizing property, prevents decomposition, while the paste remains moist. In cases where the powder is washed away by profuse oozing, the dusting may have to be repeated every half hour until the object, viz., the formation of a dry crust, is established. Care should be taken with old horses, as the too free and long-continued use of iodoform is apt to produce iodoform poisoning ; equal parts of iodoform and boracic acid are safer.

Preparations of Dressings.—Gauze, called in the trade cheeze cloth, or tobacco cloth, forms a good material for wound dressing ; it is cheap, can be bought almost anywhere, absorbs well and is soft and pliant. The raw gauze is treated as follows : Boil twenty-four yards for an hour in a wash kettle filled with sufficient water to cover the material, to which add two pounds of washing soda, or a pint of strong lye ; afterward thoroughly swill in clear water, pass through a clothes wringer, then immerse in a 1 to 1000 solution of corrosive sublimate for twenty-four hours ; pass again through a clothes wringer, dry and put away in covered jars until required for use.

Iodoformized Gauze.—The moist gauze is evenly sprinkled with iodoform powder from a pepper box, well rubbed into the meshes by hand, then put away in wide-mouthed bottles.

